# Randomised controlled trial of video clips and interactive games to improve vision in children with amblyopia using the I-BiT system

**DOI:** 10.1136/bjophthalmol-2015-307798

**Published:** 2016-03-07

**Authors:** Nicola Herbison, Daisy MacKeith, Anthony Vivian, Jon Purdy, Apostolos Fakis, Isabel M Ash, Sue V Cobb, Richard M Eastgate, Stephen M Haworth, Richard M Gregson, Alexander JE Foss

**Affiliations:** 1 Department of Ophthalmology, Nottingham University Hospitals, Nottingham, UK; 2 Department of Ophthalmology, Addenbrooke's Hospital, Cambridge, UK; 3 Department of Computer Science, University of Hull, Hull, UK; 4 Derby Clinical Trials Unit, College of Health and Social Care, University of Derby, Derby, UK; 5 Department of Mechanical, Materials and Manufacturing Engineering, University of Nottingham, Nottingham, UK; 6 Department of Ophthalmology, Nottingham University Hospitals, Nottingham, UK*

**Keywords:** Child health (paediatrics), Treatment other, Vision

## Abstract

**Background:**

Traditional treatment of amblyopia involves either wearing a patch or atropine penalisation of the better eye. A new treatment is being developed on the basis of virtual reality technology allowing either DVD footage or computer games which present a common background to both eyes and the foreground, containing the imagery of interest, only to the amblyopic eye.

**Methods:**

A randomised control trial was performed on patients with amblyopia aged 4–8 years with three arms. All three arms had dichoptic stimulation using shutter glass technology. One arm had DVD footage shown to the amblyopic eye and common background to both, the second used a modified shooter game, Nux, with sprite and targets presented to the amblyopic eye (and background to both) while the third arm had both background and foreground presented to both eyes (non-interactive binocular treatment (non-I-BiT) games).

**Results:**

Seventy-five patients were randomised; 67 were residual amblyopes and 70 had an associated strabismus. The visual acuity improved in all three arms by approximately 0.07 logMAR in the amblyopic eye at 6 weeks. There was no difference between I-BiT DVD and non-I-BiT games compared with I-BiT games (stated primary outcome) in terms of gain in vision.

**Conclusions:**

There was a modest vision improvement in all three arms. Treatment was well tolerated and safe. There was no difference between the three treatments in terms of primary stated outcomes but treatment duration was short and the high proportion of previously treated amblyopia and strabismic amblyopia disadvantaged dichoptic stimulation treatment.

**Trial registration number:**

NCT01702727, results.

## Introduction

The mainstay of treatment for amblyopia caused by anisometropia or strabismus, after correction of refractive error, is occlusion, by patching, of the normal eye. This idea of ‘forcing’ the amblyopic eye to see dates at least as far back as de Buffon.^1^ There is a sensitive period for the development of the visual system during which patching is effective. It is more effective for younger children but a randomised control trial of patching has shown that about half of children with amblyopia in the 7–12 year age range will respond to optical treatment and patching.^2^ Some children over the age of 12 years also improved but only if they had not received previous treatment.^2^

The additive effect of patching after spectacle adaptation is modest. A randomised control trial of patching in children who had 16 weeks of full refractive correction gained a further 1.3 lines in the control group (spectacle correction alone can continue to improve vision for up to 30 weeks) and 2.2 lines in those who had 2 h of patching per day.^3^ The effectiveness of patching depends upon the degree of amblyopia with little benefit for those with mild levels of amblyopia (6 September–6 December).^4^ In the 1850s, Javal introduced atropine penalisation which is as effective as patching^5–8^ and the two treatments are not additive—nothing is to be gained by combining them.^9^

Current treatments are only moderately effective with amblyopia persisting beyond the age of 16 years in 80% of patients.^10^ A further drawback shared by both patching and penalisation is that they are ‘dissociative’ and do nothing to encourage the amblyopic and the normal eye to work in harmony together. Furthermore, compliance with patching is often poor with up to 50% of patients not complying with the prescribed dosage,^11^ suggesting that the treatment is not popular with children. There is clearly a need for something better.

These considerations led us to develop a virtual reality-based system^12^ to treat amblyopia using dichoptic stimulation in the context of either playing special video games or watching DVDs. This interactive binocular treatment (I-BiT) system uses specially configured software to preferentially stimulate the amblyopic eye without compromising the vision in the good eye.

Three pilot studies^13–15^ have shown the I-BiT system can improve the visual acuity in patients with amblyopia. The most recent of these pilot studies using shutter glasses technology^15^ showed that all patients who completed their planned treatment (9 of the 10 patients) showed an improvement in visual acuity from 0.025 to 0.45 logMAR units with a mean of 0.18 (SD 0.14).

On the basis of these results, we decided to proceed to a small two centre randomised control trial with the intention of ensuring that the treatment was safe, acceptable and to get an improved indication of its efficacy.

## Materials and methods

### Trial design

The full trial design and protocol is available from a previous publication.^16^

The study was a randomised parallel group design with the intention to recruit 75 patients. The eligible patients were randomised to one of three treatments.

- I-BiT game
- Non-I-BiT game
- I-BiT DVD.

Each received their randomised treatment weekly for 6 weeks, for a 30 min period.

At baseline their logMAR visual acuity, with glasses if required, was recorded along with the results of their cover test, oculomotility assessment, binocular vision assessment, visuoscope and Sbisa bar results.

Visual acuity was assessed pretreatment (week 1), after three treatments (week 3), after six treatments (week 6) and 4 weeks after their final treatment (week 10). Visual acuity was assessed with either the logMAR crowded test (formally Glasgow acuity cards, manufactured by Keeler) or the crowded Kay's picture test. Choice of visual acuity (VA) test was dependent on the participant's ability and remained consistent throughout the trial.

### Participants and recruitment

Children aged 4–8 years with strabismic, anisometropic or mixed amblyopia were recruited from two test sites (Queen's Medical Centre, Nottingham and Addenbrooke's Hospital, Cambridge), from January 2012 to November 2013. Stimulus deprivation amblyopia was excluded.

Children who had prior treatment with either patching or atropine penalisation were eligible for recruitment providing they had a 0.20 logMAR intraocular acuity difference and no current improvement with patching. Those whose suppression measured four or less on the Sbisa bar were deemed at risk of double vision and were excluded. For full list of inclusion and exclusion criteria, see the protocol paper.^16^

### Interventions

#### I-BiT shutter glasses system

The I-BiT system hardware is designed for use under supervision and consists of a desktop PC with two monitors, one for the clinician and one for the patient. The clinician monitor is used to control the treatment the patient receives and the patient monitor displays the visual stimuli. The patient monitor is a flat-screen 22-inch 3D monitor with a refresh rate of 120 Hz and this allows separate images to be presented to each eye with the use of shutter glasses. The shutter glass lenses lighten and darken in synchrony with the monitor but faster than the user can perceive and this allows a common background to be presented to both eyes and an ‘enriched’ image to be presented only to the amblyopic eye (dichoptic stimulation). The I-BiT system can display video footage and interactive games. A gaming control pad is used for the games.

#### DVD stimulus

The I-BiT DVD stimulus is divided into two zones. There is an outer ‘border’ termed a locking stimulus which is presented to both eyes while the inner part of the screen presents the video footage predominately to the amblyopic eye.

#### Game stimulus

An interactive game called ‘Nux’ was used to provide the game play. Through the I-BiT system, the player and the background are shown to both eyes but the obstacles, enemies and coins are shown only to the amblyopic eye. Therefore, in order for the child to play the game successfully, they must use their amblyopic eye.

#### Control stimulus

In the non-I-BiT game version (control arm) both eyes receive identical stimulation.

### Outcomes

The primary objectives of the study were:

1. To determine the difference in visual acuity improvement in patients treated with I-BiT compared with non-I-BiT treatment.
2. To determine the difference in effectivity between the interactive games and DVDs.

The primary outcome measure was the change in logMAR visual acuity from week 1 (pretreatment) to week 6 (post-treatment).

The secondary outcome measures included changes in stereoacuity (Frisby test), and the safety, acceptability and compliance of treatment.

Loss of visual acuity of 0.1 logMAR units at week 3 or double vision induced withdrawal from the trial.

### Sample size

The sample size of 75 patients (25 in each arm) was calculated assuming an SD of 0.25 with the aim to detect a minimum difference of 0.2 logMAR units at the 5% significance level (two-sided) with 80% power.

### Randomisation

The randomisation sequence was generated using the RALLOC function in Stata V.10 and used random permuted blocks of sizes 2, 4 and 6. It was stratified by centre and whether or not the patient had previous treatment for amblyopia.

### Masking

The research orthoptist who delivered the treatment was aware of the patient allocation but not of visual acuity measurements which were performed by an independent orthoptist who was masked to the treatment allocation.

### Statistical methods

Data analysis was performed using Stata V.11.2. Intention to treat analysis was performed for the primary outcome. All missing data were assumed to be missing completely at random and imputation methods were not used to compensate for missing data.

The primary endpoint of change in visual acuity from week 1 to end of treatment (week 6) was compared between the treatment groups using Analysis of Covariance (ANCOVA) with baseline visual acuity as covariate. The change in visual acuity from week 1 to week 3 and 10 was compared between the treatment groups using ANCOVA with baseline visual acuity as covariate. Difference within groups from baseline to 3, 6 and 10 weeks post-treatment was tested for using a two-tailed paired t test. The proportion of patients showing a clinically important change in VA (≥0.125 logMAR) from baseline at 3, 6 and 10 weeks was compared between the three groups using Fisher's exact test. The frequency (%) of patients’ satisfaction, compliance with treatment and adverse events were also analysed.

Unplanned secondary analyses of the change in VA for all participants from baseline to weeks 3, 6 and 10 were performed using a paired t test.

### Ethical review

This study was approved by Nottingham Research Ethics Committee 2.

## Results

### Participant flow and baseline data

**Figure 1:**
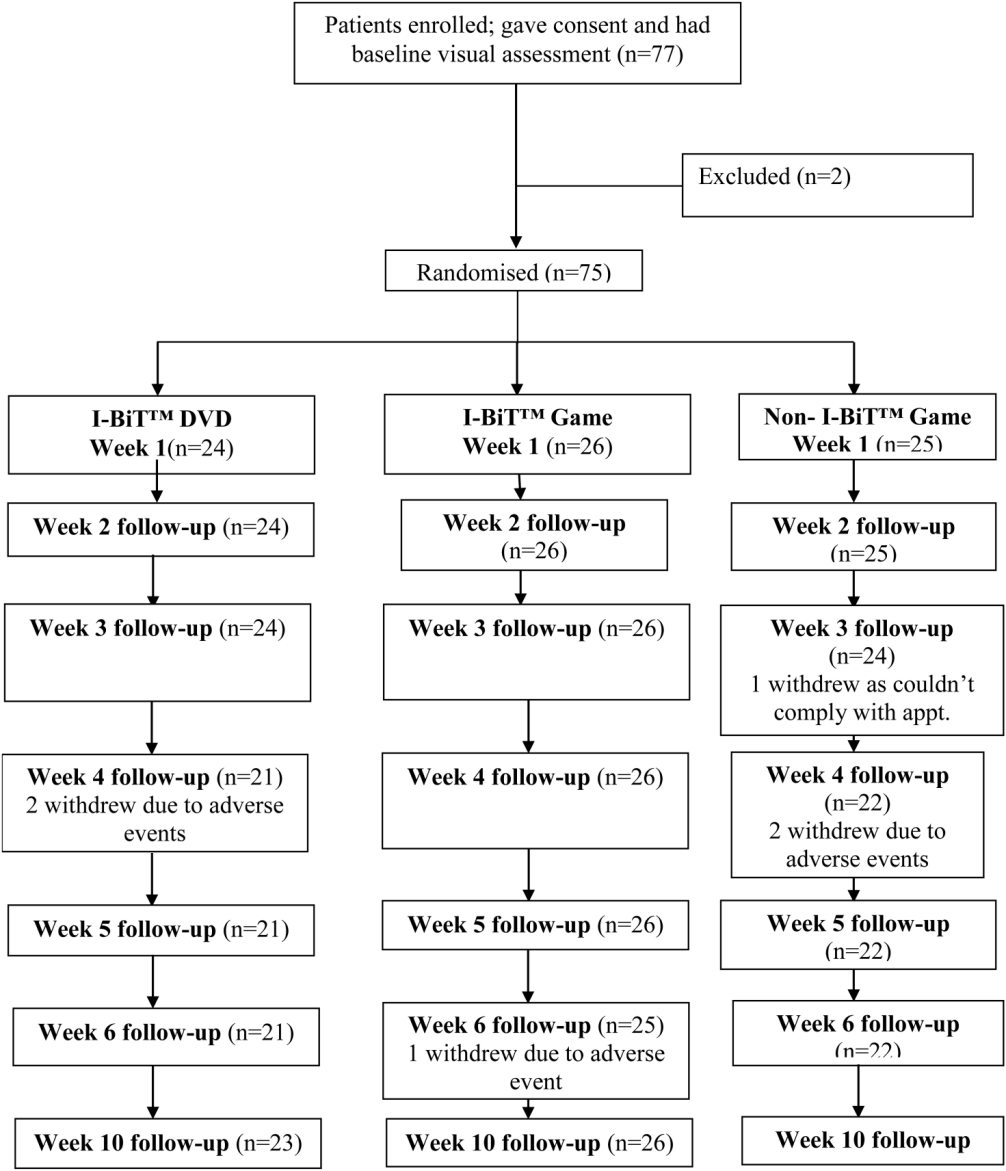
The Consolidated Standards of Reporting Trials (CONSORT) flow diagram for the trial.

The patient flow is summarised in the Consolidated Standards of Reporting Trials (CONSORT) diagram (figure 1) and the baseline data in table 1 and further baseline data can be found in the online supplementary file.

**Table 1:** Summary of baseline characteristics of the study population

	I-BiT DVD	I-BiT games	Non-I-BiT games
Number randomised	24	26	25
Mean (SD) age (years)	5.9 (1.2)	6.0 (1.3)	5.6 (1.1)
Gender			
Male (%)	13 (54%)	17 (65%)	13 (52%)
Female (%)	11 (46%)	9 (35%)	12 (48%)
Experience with computer games			
Does not play	3	5	4
<30 min/day	12	7	12
30–60 min/day	6	10	4
1–2 h/day	2	4	3
>2 h/day	1	0	2
Type of amblyopia			
Strabismic	13	6	5
Mixed	11	17	18
Anisometropic	0	3	2
Previous amblyopia treatment			
Yes	19	20	18
Occlusion	19	20	17
Penalisation	4	3	7
None	5	6	7
Acuity in amblyopic eye at baseline (logMAR)			
Mean	0.53	0.49	0.50
Range	0.36–0.65	0.38–0.55	0.35–0.65
SD	0.21	0.17	0.20
Fixation with amblyopic eye			
Foveolar	3	1	2
Parafoveolar	5	11	4
Parafoveal	8	5	2
Eccentric	4	1	2
No view with amblyoscope	4	8	15

10.1136/bjophthalmol-2015-307798.supp1 Supplementary data

The visual acuity improved in all three groups at weeks 3, 6 and 10 with the average improvement of vision at week 6 of 0.07 logMAR which was sustained at week 10. This improvement was significant at all three time points (using paired t tests, p<0.001 at all three time points) (table 2).

**Table 2:** Improvement in vision for all the patients in the trial

	Improvement in logMAR vision	CIs	p Value
Baseline and week 3	−0.04	−0.63 to −0.20	<0.001
Baseline and week 6	−0.07	−0.11 to −0.047	<0.0001
Baseline and week 10	−0.067	−0.097 to 0.038	<0.0001

Significance testing was performed using a paired t test and probability values calculated on the basis of two-tailed.

### The primary outcome

The primary outcome measure was the difference in visual acuity improvement between the three arms at week 6 with I-BiT games as the comparison arm. The change in visual acuity from baseline to the end of treatment (week 6) is shown in table 3.

**Table 3:** Summary of outcomes

	I-BiT DVD	I-BiT games	Non-I-BiT games
Number randomised	24	26	25
*Primary outcome*			
Change from baseline to week 6 in visual acuity (logMAR)			
N	21	25	22
Mean (SD)	−0.1 (0.02)	−0.06 (0.02)	−0.03 (0.02)
Median (range)	−0.1 (−0.15, 0.05)	−0.05 (−0.13, 0)	−0.04 (−0.1, 0.04)
Difference from I-BiT games			
Mean (SE)	0.05 (0.03)		0.02 (0.03)
95% CI	−0.004 to 0.10		−0.07 to 0.03
p Value (from ANCOVA with baseline VA as covariate)	0.067		0.429
*Secondary outcomes*			
Change in VA from baseline to week 10			
Change from baseline to week 10 in visual acuity (logMAR)			
N	23	26	24
Mean (SE)	−0.07 (0.03)	−0.07 (0.03)	−0.06 (0.02)
Median (IQR)	−0.08 (−0.18, −0.02)	−0.09 (−0.16, −0.07)	−0.05 (−0.15, 0)
Difference from I-BiT games			
Mean (SE)	0.003 (0.04)		−0.01 (0.04)
95% CI	−0.07 to 0.07		−0.08 to 0.06
Change in VA from baseline to week 3			
Change from baseline to week 3 in visual acuity (logMAR)			
N	24	26	24
Mean (SD)	−0.05 (0.02)	−0.05 (0.02)	−0.02 (0.02)
Median (IQR)	0.07 (−0.12, −0.005)	−0.04 (−0.1, 0.008)	−0.03 (−0.08, 0.02)
Difference from I-BiT games			
Mean (SE)	0.002 (0.03)		−0.03 (0.02)
95% CI	−0.06 to 0.06		−0.08 to 0.01
Proportion (%) of patients showing a clinically important change in VA (≥1.25 logMAR)			
Week 3	6 (25%)	4 (15%)	1 (4%)
Week 6	6 (25%)	6 (23%)	4 (16%)
Week 10	10 (42%)	11 (42%)	8 (32%)

There was no difference in the change from baseline between those having I-BiT games and those having non-I-BiT games (mean 0.02 logMAR units 95% CI (−0.07 to 0.03)). The improvement in vision from baseline at 6 weeks differed between the two games treatment groups and the DVD group, with a mean of change in the I-BiT DVD group of −0.1 compared with −0.06 in the I-BiT games group and −0.03 in the non-I-BiT games group.

### Secondary outcomes

The change in vision at weeks 3 and 10, and the proportion of patients with a clinically significant improvement (defined as ≥1.25 logMAR units) are summarised in table 3.

There were no significant improvements in stereoacuity (Frisby) in the three arms (see online supplementary material).

The I-BiT DVD and non-I-BiT game improved vision to week 6 then declined slightly after treatment finished (week 10). The I-BiT games group increased from weeks 3–6 and then a decrease to week 10. The mean VA at week 10 was less than baseline for all three groups.

The patient satisfaction questionnaire showed that >90% of participants felt that they enjoyed their treatment, >80% felt that the time allowed was just right, and that 67% or more felt that it was easy to concentrate.

Compliance with each of the treatments was excellent with the majority of participants playing the game/watching the DVD for 30 min at each session.

### Adverse events

These are summarised in table 4. There were two cases of double vision which were assumed to be adverse device effects and they both resolved spontaneously following cessation of treatment. All other adverse events are assumed to be not device related.

**Table 4:** Summary of adverse events in the trial

	I-BiT DVD N=24	I-BiT game N=26	Non-I-BiT game N=25
Adverse events leading to withdraw from trial			
Double vision	1	1	0
Drop in vision	2	0	2
Adverse events not leading to withdraw			
Flu or cough	2	2	2
Tonsillitis	1	0	1
Conjunctivitis	1	0	
Diarrhoea and/or vomiting	0	6	2
Other infection	0	1	0
Eczema	1	0	1
Trauma	1	0	1
Eye feeling funny	1	0	0

## Discussion

There was a modest visual acuity improvement in all groups including group 3 who received the non-I-BiT game. The visual acuity improvement of 0.07 logMAR is less than the 0.18 which we had observed previously but is similar to the 0.1 improvement reported by Li *et al*.^17^ It was an unexpected finding that the non-I-BiT game showed some improvement in visual acuity and the reason is not clear. It is possible that use of the shutter glasses alone may have improved amblyopic vision. The control group had all of the game content shown to both eyes but because of the shutter glasses it is, in reality, only shown to one eye at a time. The shutter glasses operate fast enough that the game content is perceived by both eyes simultaneously. Dichoptic stimulation may itself improve vision without the use of ‘preferential stimulation’ of the amblyopic eye which occurred in the I-BiT game and I-BiT DVD groups.

The study was disadvantaged by the fact that the majority of participants in this trial (67/75) were residual amblyopes (conventional patching failures) and there was a high proportion of strabismic amblyopia (70/75). This represents a poor prognostic group but the decision to allow previous amblyopia treatment failures into the trial was based on the fact that they were far easier and quicker to recruit. Another limiting factor was that the treatment in this trial was hospital based during working and school hours which limited the duration and frequency of treatment sessions to only half an hour a week. The total treatment time of only 3 h is very short when compared with patching where the Monitored Occlusion Treatment of Amblyopia (MOTAS) group recommended at least 150 h and showed a linear response for up to 400 h.^18^ The study by Li *et al* used an I-Pad delivery system with 16 h of game play >4 weeks and with no extra benefit from a further 16 h over a further 4 week period.^17^ Hess's group, using an I-Pad at home^19^ showed that 1–2 h per day for 1–3 weeks can improve acuity, restore binocularity and restore stereopsis in adults.^20^ Vedamurthy *et al* also developed a dichoptic action videogame and with 40 h of treatment in adults showed improvements in both anisometropic and strabismic amblyopes of logMAR 0.14.^21^

There were two classes of adverse events that lead to patient withdrawal diplopia and reduction visual acuity Double vision is presumed to be due to the treatment reversing suppression and was considered a device-related adverse event and the two cases resolved fully on cessation of treatment. No adverse events have been reported by other groups.

Patching and penalisation are not popular treatments and systematic reviews have suggested that the impact on health-related quality of life for patients with amblyopia comes from the treatment rather than the disease.^22^ ^23^ This dichoptic stimulation treatment was very acceptable and with a compliance rate of >90%, much better than the 48% reported for patching.^24^ Others have also noted the high acceptability of computer games as a vehicle for treatment.^25^ Amblyopia often coexists with strabismus and 70 of the 75 patients in this trial had manifest strabismus. Failure of the amblyopic eye to become the fixating eye may prevent dichoptic stimulation being an effective treatment in patients with strabismus unless the stimulus can be delivered to the fovea.

There was some improvement in vision in all three groups after a total of 3 h of treatment which was well tolerated and safe. There was no apparent advantage to differential targeting of the amblyopic eye with dichoptic stimulation. However, the short total treatment time, high proportion of previous amblyopia treatment failures and high proportion of patients with strabismus disadvantage dichoptic stimulation and this study does not suggest that a treatment benefit is not achievable. Further studies are being targeted at longer treatment duration (requiring a home-based treatment), naïve amblyopes and a greater proportions of anisometropic amblyopes to determine whether the I-BiT system has the potential to deliver a treatment for amblyopia which is more acceptable and equally or more effective than conventional treatment.
