# Supplementary material for: Randomised controlled trial of video clips and interactive games to improve vision in children with amblyopia using the I-BiT system
Source: Br J Ophthalmol. 2016 Mar 7;100(11):1511–6. doi: 10.1136/bjophthalmol-2015-307798 (PMC5136691; doi:10.1136/bjophthalmol-2015-307798)
Supplement: Supplementary data [file bjophthalmol-2015-307798supp.pdf]

Supplementary Material for the I-BiT Trial

Pre-treatment Prism Cover Test on an intention to treat basis for the study population at baseline.

	I-BiT DVD	I-BiT Games	Non I-BiT Games	**Total**
N randomised	24	26	25	75
Test				
Prism cover test	24(100%)	26(100%)	25(100%)	75(100%)
Prism reflection test	0(0%)	0(0%)	0(0%)	0(0%)
Test performed with glasses				
Yes	23(96%)	25(96%)	25(100%)	73(97%)
No	1(4.2%)	1(3.9%)	0(0%)	2(2.7%)
Prism cover test				
Inconclusive	1	1	1	3
Near				
N	23	26	22	71
0-<10	10(43%)	15(58%)	13(59%)	38(54%)
10-<20	7(30%)	5(19%)	4(18%)	16(23%)
20-<30	5(22%)	5(19%)	3(14%)	13(18%)
30-< 40	0(0%)	0(0%)	1(4.54%)	1(1.41%)
40-<50	1(4%)	0(0%)	3(14%)	4(6%)
>50	1(4.4%)	1(3.9%)	1(4.5%)	3(4.2%)
ET	15(65%)	14(54%)	13(%)	42(59%)
XT	1(4.4%)	2(7.7%)	3(%)	6(8.5%)
X	0(0%)	5(19%)	3(%)	8(11%)
E	5(227%)	3(12%)	4(%)	12(17%)
Distance				
N	24	26	25	75
0-<10	13(54%)	19(73.08%)	15(60%)	47(62.67%)
10-<20	7(29%)	6(23.08%)	3(12%)	16(21.33%)
20-<30	3(14%)	0(0%)	6(24%)	9(12%)
30-< 40	0(0%)	0(0%)	0(0%)	0(0%)
40-<50	0(0%)	0(0%)	0(0%)	0(0%)
>50	1(4.17%)	1(3.85%)	1(4%)	3(4%)
ET	14(58%)	14(54%)	13(52%)	41(55%)
XT	1(4.2%)	2(7.7%)	4(16%)	7(9.33%)
X	0(0%)	5(19.23%)	4(16%)	9(12%)
E	4(17%)	3(12%)	2(8%)	9(12%)

Pre-treatment Binocular Functions on an intention to treat basis for the study population

	I-BiT DVD	I-BiT Games	Non I-BiT Games	**Total**
N randomised	24	26	25	75
**Bagolini Glasses/Worth's lights**				
BSV				
Near	13(54%)	19(73%)	15(60%)	47(63%)
Distance	12(50%)	16(62%)	15(60%)	43(57%)
Suppression (right)				
Near	4(17%)	0(0%)	2(8%)	6(8%)
Distance	6(25%)	2(7.7%)	2(8%)	10(13%)
Suppression (left)				
Near	1(4.17%)	4(15.38%)	2(8%)	7(9.33%)
Distance	3(13%)	7(27%)	4(16%)	14(19%)
Suppression (alt)				
Near	0(0%)	0(0%)	0(0%)	0(0%)
Distance	0(0%)	0(0%)	0(0%)	0(0%)
Diplopia				
Near	1(4.2%)	0(0%)	0(0%)	1(1.3%)
Distance	0(0%)	0(0%)	0(0%)	0(0%)
Inconclusive				
Near	5(21%)	3(12%)	6(24%)	14(19%)
Distance	3(13%)	1(3.9%)	4(16%)	8(11%)
**Frisby**				
Seconds of arc				
N	11	11	12	34
Mean (sd)	331(224)	195(170)	231(151)	252(187)
Median (range)	300(110,600)	170(55,300)	193(101,340)	170(108,340)
Inconclusive				

Changes in Prism Cover test from Baseline to Week 10 on an intention to treat basis for the study population

Near	Baseline			10 weeks		
	I-BiT DVD	I-BiT Game	Non I-BiT Game	I-BiT DVD	I-BiT Game	Non I-BiT Game
Prism Cover N<br>Mean (sd)<br><br>Median (IQR)	23<br>12.35<br>(10.39)<br>12<br>(4, 20)	25<br>8.04<br>(7.85)<br>4<br>(1.5, 15)	24<br>13.67<br>(14.03)<br>7.5<br>(4.5, 20)	22<br>10.91<br>(9.17)<br>9<br>(3, 20)	26<br>8.31<br>(7.49)<br>6<br>(2.8, 13)	24<br>12.5<br>(12.22)<br>7.5<br>(4, 19)
Change from baseline<br>N<br>Mean (sd)<br><br>Median (IQR)	NB empty as change from baseline			22<br>-0.95<br>(4.24)<br>0<br>(-2.5, 1.25)	25<br>0.48<br>(3.03)<br>1<br>(0, 2)	23<br>-1.04<br>(6.26)<br>0<br>(-3, 2)
**Distance**	**Baseline**			**10 weeks**		
	I-BiT DVD	I-BiT Game	Non I-BiT Game	I-BiT DVD	I-BiT Game	Non I-BiT Game
Prism Cover N<br>Mean (sd)<br><br>Median<br>(IQR)	23<br>9.13<br>(7.85)<br>8<br>(2, 15)	25<br>5.72<br>(5.70)<br>4<br>(0.5, 9)	24<br>9.58<br>(9.55)<br>7<br>(1.25, 19)	22<br>8.77<br>(9.45)<br>6<br>(2, 12.75)	26<br>7.15<br>(5.41)<br>8<br>(2, 12)	24<br>10.25<br>(10.41)<br>6<br>(2.25, 18)
Change from baseline<br>N<br>Mean<br>(sd)<br>Median<br>(IQR)	NB empty as change from baseline			22<br>-0.09<br>(4.93)<br>0<br>(-4, 2)	25<br>1.16<br>(2.91)<br>1<br>(0, 3)	23<br>0.78<br>(4.46)<br>0<br>(-1, 4)

Binocular functions on an intention to treat basis

	Baseline			Week 10		
	I-BiT DVD	I-BiT Games	Non I-BiT Games	I-BiT DVD	I-BiT Games	Non I-BiT Games
N	24	26	25	23	26	24
**Bagolini Glasses/Worth's lights**						
BSV						
Near	13(54%)	19(73%)	15(60%)	10(43%)	16(62%)	15(63%)
Distance	12(50%)	16(62%)	15(60%)	9(39%)	11(42%)	14(58%)
Suppression (right, left and alt)						
Near	5(21%)	4(15%)	4(16%)	93(9.1%)	6(24%)	6(25%)
Distance	9(38%)	9(35%)	6(24%)	9(393%)	11(42%)	7(29%)
Diplopia						
Near	1(4.2%)	0(0%)	0(0%)	0(0%)	0(0%)	0(0%)
Distance	0(0%)	0(0%)	0(0%)	0(0%)	0(0%)	0(0%)
Inconclusive						
Near	5(21%)	3(12%)	6(24%)	4(17%)	4(15%)	3(13%)
Distance	3(13%)	1(3.9%)	4(16%)	5(21%)	4(15%)	3(13%)
**Synoptophore**						
Angle of anomaly						
Yes	9(38%)	7(27%)	4(16%)	5(22%)	4(15%)	3(13%)
No	2(8.3%)	3(12%)	2(8%)	1(4.4%)	3(11%)	2(8.3%)
Inc	4(17%)	4(15%)	8(32%)	9(39%)	5(19%)	8(33%)
NA	9(38%)	12(46%)	11(44%)	8(35%)	14(54%)	11(46%)
Potential for fusion						
Yes	6(25%)	4(15%)	5(20%)	2(8.7%)	2(7.7%)	2(8.3%)
No	6(25%)	8(31%)	6(24%)	8(35%)	6(23%)	7(29%)
Inc	3(13%)	2(7.7%)	3(12%)	4(17%)	1(3.9%)	4(17%)
NA	9(38%)	12(46%)	11(44%)	9(39%)	17(65%)	11(46%)
Potential for stereopsis						
Yes	4(17%)	3(12%)	1(4%)	2(8.7%)	1(3.9%)	2(8.3%)
No	8(33%)	8(30.77%)	7(28%)	9(39%)	5(19%)	7(29%)
Inc	3(13%)	3(12%)	6(24%)	3(13%)	3(13%)	4(17%)
NA	9(38%)	12(46%)	11(44%)	9(39%)	9(39%)	11(46%)

Changes in Frisby from Week 1 to Weeks 3, 6 and 10: Intention to treat population

	Week 1			3 weeks			6 weeks			10 weeks		
	I-BiT DVD	I-BiT Games	Non I-BiT Games	I-BiT DVD	I-BiT Games	Non I-BiT Games	I-BiT DVD	I-BiT Games	Non I-BiT Games	I-BiT DVD	I-BiT Games	Non I-BiT Games
Frisby												
N	11	13	11	10	15	11	10	15	12	10	13	12
Mean	181	166	116	173	191	153	125	186	156	104	92	113
(sd)	(104)	(1499)	(68)	(93)	(161)	(156)	(85)	(153)	(102)	(26)	(44)	(81)
Median	150	110 (110,	110	150	110	110	98	110	110	98	85	85
(IQR)	(85, 300)	160)	(40, 170)	(104, 236)	(85, 340)	(55, 170)	(70, 150)	(85, 340)	(85, 215)	(85, 110)	(55, 118)	(55, 140)
N negative/inc	13	13	14	14	11	13	11	10	10	13	13	12
Missing	0	0	0	0	0	1	3	1	3	1	0	1
Change from baseline	NB empty as change from baseline											
N				10	13	11	10	12	11	10	11	11
Mean				-9	-18	36	-58	178	23	-79	-47	-8.2
(sd)				(42)	(100)	(125)	(107)	(169)	(73)	(99)	(82)	(111)
Median				0	0	0	-28	-5	0	-65	-35	-25
(IQR)				(0, 0)	(-70, 10)	(0, 55)	(-135, 14)	(-55, 49)	(-25, 60)	(-155, 7.5)	(-75, 0)	(-85, 15)
Missing				0	0	1	3	1	3	1	0	1
Changed to negative				0	0	0	0	0	0	0	0	0
Changed from negative				0	0	0	0	0	0	0	0	0
